# Compare and evaluate fracture strength of endodontically treated mandibular premolar teeth restored using different fiber post and core systems: An *in vitro* study

**DOI:** 10.6026/973206300221540

**Published:** 2026-03-31

**Authors:** Sai Tejaswi Devarapalli, Bharathi Munagapati, Y Poojitha1, Challa Deepika, Saroja Amaladinna, Sreelekha Cigicherla

**Affiliations:** 1 Department of Prosthodontic Crown and Bridge, Narayana Dental College and Hospital, Nellore, Andhra Pradesh, India; 2 Department of Prosthodontics Crown and Bridge, G. Pulla Reddy Dental College and Hospital, Kurnool, Andhra Pradesh, India

**Keywords:** Endodontically treated teeth, post and core, glass fiber post, carbon fiber post, fracture strength

## Abstract

The optimal method for restoring endodontically treated teeth remains a subject of ongoing clinical debate. While prefabricated posts are frequently recommended to support final restorations, their necessity and overall effectiveness continue to be a point of professional controversy. Therefore, it is of interest to evaluate the effect of fiber posts on the fracture resistance of endodontically treated mandibular premolars, where 60 standardized mandibular premolars were restored using and divided into three groups: Group A (no post), roup B (glass fiber post) and Group C (carbon fiber post). A universal testing machine was used to apply axial force and determine the failure point of each specimen. Thus, we show that the highest fracture strength is maintained when teeth are restored without a post, provided sufficient natural tooth structure is preserved. In cases where a post is required, glass fiber systems demonstrated statistically significant superiority over carbon fiber alternatives. Consequently, the study concludes that posts do not reinforce the tooth structure but are primarily for restoration retention, with glass fiber being the preferred material for its strength and aesthetic properties.

## Background:

In addition to replacing missing teeth, prosthodontics aims to preserve endodontically treated teeth by providing appropriate restorations that promote optimal function and appearance while shielding them against failure and subsequent infection. Endodontic therapy removes tooth vitality by extracting pulpal tissue, resulting in a less hydrated, more brittle tooth prone to fractures under chewing forces. Therefore, treatment planning must consider factors that strategically strengthen the non-vital tooth [1]. Non-vital teeth are prone to fracture under vertical and lateral occlusal stresses, making restoration challenging due to significant structural loss. Success depends on selecting modern prosthodontic or endodontic materials and techniques tailored to each tooth's diagnosis [2]. Significant loss of coronal tooth structure often requires a core build-up to retain the final prosthodontic restoration. In many cases, an intra-radicular post is also needed to support and retain the core material [3]. The primary cause of failure in endodontically treated teeth is often the inappropriate selection of restorative techniques. Factors such as the amount of remaining tooth structure, the tooth's position in the arch, functional demands and aesthetic requirements should all be considered when choosing the most suitable restorative approach [4]. A variety of techniques and materials have been developed for restoring endodontically treated teeth [5]. These include traditional metallic options such as prefabricated and cast metal posts, as well as modern non-metallic alternatives. Among the latter, epoxy resin posts reinforced with materials like carbon fibers, zirconia, glass fibers and polyethylene fibers have become increasingly popular due to their favorable strength, esthetics and compatibility with tooth structure [6]. Fiber post is a relatively recent development in the restorative dentistry. The unequal distribution of stress between the post and teeth due to the metal post's modulus of elasticity caused numerous and frequent dislodgments or in some cases even tooth fractures, which ultimately led to the failure of the metal post system had a decline in preference. At the same time, the use of fiber posts has grown in popularity. Numerous investigations on the fiber post and its various features have been published in their literature [7 8-9]. Due to the large number of commercially available fiber posts now on the market, the current study was developed to investigate the fracture strength of mandibular premolars that were endodontically treated and restored using two distinct fiber post and core systems.

## Materials and Methodology:

A total of 60 extracted human maxillary molars were used in this study (Figure 1 - see PDF). The teeth were examined for any prior restorations, fractures and cracks which were randomly divided into 3 groups.

## Standardization of samples:

In order to standardize each sample first teeth were stored in artificial saliva (WET MOUTH, ICPA health product ltd, India) except during restoration and experimental testing. After which all the teeth were decoronated to a length of 15mm (Figure 2 - see PDF) from apex of teeth 2mm from cemento enamel junction were removed using diamond disc mounted to micro motor hand piece (MARATHON, SAE yang co, Korea) and these sectioned teeth were mounted into acrylic blocks.

## Grouping of samples:

[1] GROUP -A: In these 20 endodontically treated mandibular premolars are not subjected to any of the post and core procedures which act as positive control group.

[2] GROUP -B: In these 20 endodontically treated mandibular premolars are restored with metal ceramic crowns using glass fiber posts (Milyard glass fiber post).

[3] GROUP -C: In these 20 endodontically treated mandibular premolars are restored with metal ceramic crowns using carbon fiber posts (Angelus carbon fiber post).

## Standardization of endodontic treatment:

Every tooth in each group was subjected to root canal instrumentation, cavity preparation, ethylenediaminetetraacetic acid (Waldent 17% EDTA) and sodium hypochlorite irrigation (PRIME 3% sodium hypochlorite) and warm vertical compaction obturation using gutta-percha (SURE- ENDO, Korea) along with sealerzinc oxide eugenol (PRIME dental products l td, Maharashtra, Mumbai, India)

## Post and core placement:

Following endodontic therapy, the group-allocated post and core system were followed a standard canal diameter and depth were suggested by the manufacturer of each post system. Using which proper root canal adaptation and stability of post was obtained.

## Cementation of post:

After post space preparation prefabricated post of size#2, post length of 10 mm was determined, prepared post space was etched with and then luted using dual cure composite luting cement (PREVEST FUSION ULTRA D/C).

## Core builds up and tooth preparation:

After post placement core build up was done using light cure composite resin (IVOCLARVIVADENTTETRIC N- CERAM LIGHT CURE COMPOSITE, Austria), light cured with (WALDENT ECO PUS LIGHT CURE, India)

## Ferrule preparation:

Parallel wall of dentine extending coronal to the shoulder of the preparation. It is possible that the extension of dentine when encircled by a crown provide a protective effect by reducing stress with in a tooth. 2mm ferrule with 1mm shoulder finish line was prepared using diamond bur of head size ISO no 010 (SF 41, MANI, Japan) diamond bur.

## Wax pattern and crown fabrication:

Each crown was waxed (UNIWAX, delta labs, India) to the height of 10mm, mesiodistal width of 8.5mm and buccolingual width of 7mmwax pattern were spured and invested in Deguvestg Rimpact (DENTSPLY international company, Germany) investment according to manufacture instruction and then metal ceramic crowns were then fabricated.

## Crown placement:

The crowns were cemented using GIC luting cement (GC- GOLD LABEL, GC CORPORATION, JAPAN). All cementation procedures were kept under a constant load after complete seating until complete cement setting occurred.

## Testing of samples:

## Fracture strength testing:

Each sample which is restored accordingly was then subjected to vertical load using universal testing machine with a cross-head speed of 1.00mm/min was applied until post fracture. The value of maximum force applied was obtained in newton (N) was recorded for analysis (Figure 3 - see PDF).

## Data analysis:

Fracture strengths data were recorded and statistically analyzed. To determine group differences, statistical analysis used mean values, standard deviations and ANOVA. Tukey's multiple comparison tests was used for post-hoc group comparison analysis.

## Results:

The fracture strengths of two different fiber post and core systems were listed in the tables in a comprehensible manner. Table 1 (see PDF) displays the mean fracture resistance and standard deviation for each material with glass fiber post and core systems having maximum fracture resistance when compared with carbon fiber post and core systems. Using one way ANOVA test ,The mean and standard deviations of fracture strengths of each group (A, B, C) are listed and compared in (Table 2 - see PDF) showing a statistical significance. Further post -hoc analysis has been used to list and compare the inter group fracture strengths which are listed and compared in (Table 3 - see PDF). A bar chart has been drawn summarizing the comparative fracture strengths of three groups in (Figure 4 - see PDF).

## Discussion:

Endodontically treated teeth are prone to breaking primarily because the removal of tooth structure during surgery weakens their physical integrity and increases flexibility. Additionally, the loss of internal nerves reduces the tooth's ability to sense excessive biting pressure, making it more vulnerable to accidental damage. Consequently, selecting a strong final restoration is the most critical factor in preventing future fractures and ensuring the tooth survives [10]. Use of cast metal post and cores have been the choice for treating endodontically treated teeth with unfavorable coronal structure, which has been showing high success rate till date [11s]. Leempoel *et al.* (1987) in their studies evaluated a large sample of teeth restored with single crown and found that 39% have received some type of post restorations that reinforced the strength of endodontically treated teeth [12]. Research attention has switched to post-material post-designs, luting agents and the ferrule effect in an attempt to strengthen teeth that have undergone endodontic treatment. Despite its high strength, precise fit and excellent retention, cast metal posts and cores continue to lag behind due to their inherent high modulus of elasticity, unfavorable stress concentration and catastrophic fracture, which has led to the development of restorative techniques using various materials and techniques [13]. In the days of increasing esthetic demands routine porcelain- fused to metal crown with sub gingival margins was never concern. So, in the present day, all ceramic restorations crowns, on lays and veneers the margins are often supra gingivally placed. A metallic or the post with darker shade (carbon fiber) or core will have a "shine through" effect. Due to this main concern, several tooth-colored posts have been developed and the use of composite core build up has enhanced the shade and translucency of the natural teeth. Torbjörner *et al.* observed flex ural modulus to stainless steel post [14], whereas Isidor *et al.* in their studies concluded that, the carbon post has higher fracture strength than prefabricated titanium or metal post [15]. Sharma *et al.* conducted studies and concluded that quartz post have higher fracture resistance over carbon fiber post [16]. Sonkesriya *et al.* in their studies observed higher fracture resistance with fiber reinforced and carbon post compared to metal or custom post [17]. In the study conducted by Singala *et al.* the fractural strength of Stainless steel, cast post, glass fiber, carbon fiber was compared and concludes, that fiber posts have more fractural strength in comparison with stainless posts [18]. In present study, we found that though the fracture resistance of GROUP -A which was taken as control is higher than the GROUP -B and Group -C. when we compared GROUP -B with GROUP- C, the fracture resistance in teeth restored with glass fiber posts shown higher when compared with carbon fiber post, these esthetic posts provide sufficient fracture strength. The results showed that, the fracture strength of samples of GROUP- B restored using glass fiber has increased when compared to that of GROUP-C restored using carbon fiber. GROUP-A had a mean of 1277.285, GROUP- B had a mean of 1032.270 and GROUP -C had a mean of 899.110. There has been a significant difference amongst the groups restored with different posts to that of teeth which has not underwent any post restoration with a significance value (<0.05) and tukey HSD test signifies a value of0.005.Hi-Rem posts feature a central soft polymer core designed to guide drills for easier removal, addressing a major difficulty of standard FRC systems like D.T. Light-Post. While research as of 2026 confirms Hi-Rem has comparable bond strength, data on its specific fracture resistance and failure modes remains limited compared to established quartz fiber systems. Both systems prioritize "favorable" non-catastrophic failure modes, but Hi-Rem's primary clinical advantage is its simplified retrievability during endodontic failures [19]. As digital dentistry emerging day- to -day CAD/CAM milled fiber post are much easier to use Traditional fiber posts have fibers running in one direction, while new CAD/CAM materials use a multidirectional layout for better overall strength [20]. Study conducted by Suzaki *et al.* shows these materials are 2.5 times more resistant to breaking when the biting force hits the fibers perpendicularly [21].

## Conclusion:

We show that the fracture resistance of teeth treated with glass fiber post when compared with carbon fiber post has provided a sufficient fracture resistance to withstand occlusal loads, on contrary, the carbon fiber post being non esthetic post with comparatively lower fracture resistance. With ever increasing demand of esthetics and shorter treatment appointments prefabricated glass fiber post showed better fracture strength in comparison to the carbon fiber post. This ultimately decreased the use of technically sensitive procedures of custom-made post and core systems and their chances of failures which may occurs in each and every step of the fabrication of post.

## Limitations:

Limitation of the present study is thermal changes in oral environment and masticatory forces were not applied and it was *in vitro* study. Further, long-term *in vitro* studies are required to evaluate the fracture strengths of different esthetic post sin oral environment for successful post.
